# Neuroprotective role of delta opioid receptors in hypoxic preconditioning

**DOI:** 10.3906/sag-1810-51

**Published:** 2019-10-24

**Authors:** Şevin GÜNEY, Sibel DİNÇER, Güleser GÖKTAŞ, Gülnur TAKE-KAPLANOĞLU

**Affiliations:** 1 Department of Physiology, Faculty of Medicine, Gazi University, Ankara Turkey; 2 Department of Histology and Embryology, Faculty of Medicine, Lokman Hekim University, Ankara Turkey; 3 Department of Histology and Embryology, Faculty of Medicine, Gazi University, Ankara Turkey

**Keywords:** Hypoxic preconditioning, delta opioid receptors, neuroprotection

## Abstract

**Background/aim:**

The purpose of the present study was to explore the neuroprotective role of delta opioid receptors (DOR) in the rat cortex in hypoxic preconditioning.

**Materials and methods:**

Rats were randomly divided into 8 groups: control (C), sham (S), hypoxic preconditioning (PC), severe hypoxia (SH), PC + SH, PC + SH + Saline (PS), PC + SH + DPDPE (DPDPE, selective DOR agonist), PC + SH + NT (NT, Naltrindole, selective DOR antagonist). Drugs were administered intracerebroventrically. Twenty four h after the end of 3 consecutive days of PC (10% O2, 2 h/day), the rats were subjected to severe hypoxia (7% O2 for 3 h). Bcl-2 and cyt-c were measured by western blot, and caspase-3 was observed immunohistochemically.

**Results:**

Bcl-2 expressions in the PC group were higher than in control, SH, and PC + SH groups. Even though there were no significant differences between the groups in terms of cyt-c levels, caspase-3 immunoreactivity of cortical neurons and glial cells in the severe hypoxia and NT groups were higher than in the control, sham, and hypoxic preconditioning groups. DPDPE administration diminished caspase-3 immunoreactivity compared with all of the severe hypoxia groups.

**Conclusions:**

These results suggest that cortical cells are resistant to apoptosis via increased expression of Bcl-2 and decreased immunoreactivity of caspase-3 in the cortex, and that DOR is involved in neuroprotection induced by hypoxic preconditioning via the caspase-3 pathway in cortical neurons.

## 1. Introduction

Hypoxia can occur due to natural causes, such as travel to or living in high altitudes, or it can occur because of pathological events such as ischemia, cardiac arrest, or blood loss. Neurons of the central nervous system are particularly sensitive to different forms of hypoxia because of their high metabolic demands and limited glucose storage capacity. Hypoxia/ischemia triggers a variety of processes that lead to changes in intracellular signal transduction, membrane function, metabolism, and even cell morphology [1,2]. These processes may cause cellular damage or death. 

Hypoxic or ischemic preconditioning (PC or IPC) s based on a phenomenon in which transient and mild hypoxia/ischemia set cells and increase cellular resistance against subsequent lethal ischemic/hypoxic injury. In recent years, several studies have shown the protective effects of PC/IPC on different organs such as the kidney, lung, and heart in both in vivo and in vitro experimental settings [3–7] However, information on the underlying mechanisms of PC/IPC which provide protection against ischemic/hypoxic insults is limited, and still being investigated.

Endogenous opioid peptides function as neurotransmitters or neuromodulators in the central nervous system and exert their effects via mu, delta, and kappa receptors (MOR, DOR, and KOR, respectively). These receptors are G protein-coupled receptors (GPCR) and are common in the central nervous system [8].

Several studies have shown that among all opioid receptors, DOR is the most sensitive to stressors like hypoxia/ischemia, and it may have a role in neuroprotective processes against hypoxic/ischemic stress [9]. 

DOR has been shown to be more abundant in the turtle brain than in the rat brain; concordant with this, neurons in turtle brain are much more resistant to hypoxic/ischemic stress [10,11]. DOR expression in the rat brain cortex is more prominent compared to subcortical areas such as the thalamus, hippocampus, or brain stem [12]; in addition, the cortex is more tolerant to hypoxic stress [13–15]. DOR expression is heterogeneous in cortex tissue. Outer and inner layers of the cortex have a higher density than the middle layer and are more resistant to hypoxia/ischemia than the middle layer [15–17]. Moreover, it has been shown that a DOR ligand, enkephalin, increases during hypoxia [18–20]. Several ischemia or stroke models performed on rats have shown that DOR upregulation or administration of central and peripheral DOR agonists decreased infarct volume, neurological deficits, and neuronal loss, and increased neuronal survival particularly in the cortex and hippocampus after ischemia [21–25]. DOR signals have been shown to protect ionic homeostasis at the beginning of hypoxia [1,26,27] and to increase the expressions and functions of beneficial signal molecules, and to decrease the expression and function of molecules which are related with cellular injury and death during long-term hypoxia [9,28,29].

All of these findings suggest that DOR expression may have an important role in neuroprotective processes during hypoxic/ischemic conditions. However, most of the studies on the protective effect of DOR against hypoxic/ischemic injury via preconditioning are in vitro studies, and information about in vivo systems is limited.

Therefore, hypoxic/ischemic preconditioning-related neuroprotective effects of DOR on hypoxic/ischemic processes still need to be clarified. With the present study, we aimed to investigate in vivo the neuroprotective role of delta opioid receptors [DOR] in the rat cortex in the presence of hypoxic preconditioning.

## 2. Materials and methods

Animal experiments were performed in accordance with the guidelines issued by the Ethical Committee of Gazi University Faculty of Medicine.

The Wistar albino rats were maintained under standard animal care conditions (12:12 h light–dark cycle, room temperature 21 °C, with standard rat chow and tap water ad libitum). Experimental groups were as follows: 1. control (C); 2. sham (S); 3. hypoxic preconditioning (PC); 4. severe hypoxia (SH); 5. hypoxic preconditioning + severe hypoxia (PC + SH); 6. hypoxic preconditioning + severe hypoxia + physiological saline (PS); 7. hypoxic preconditioning + severe hypoxia + DPDPE (DPDPE, a delta opioid receptor agonist); 8. PC + SH + Naltrindol (NT, a delta opioid receptor antagonist). Number (n) of subjects in the groups were between 4 and 6 each, explained in the relevant figures in the results section. 

All rats except for those in the control group were anesthetized with ketamine (90 mg/kg) and xylazine (5 mg/kg) and then placed in a stereotaxic instrument. A 22-gauge stainless steel guide cannula was inserted into the lateral cerebral ventricle (AP: 0.8 mm, L: 1.5 mm). Animals were allowed to recover for at least 1 week before experiments.

Severe hypoxia (SH) was produced by using 7% normobaric oxygen for 3 h in a normobaric chamber. For hypoxic preconditioning, the animals were kept at 10% normobaric oxygen for 2 h daily for 3 days before SH. Before the SH bout, 25 μg DPDPE, NT, or PS was applied to the rats intracerebroventricularly.

After hypoxia protocols, the rats were anesthetized with ketamine (90 mg/kg) and xylazine (5 mg/kg) cocktail and decapitated. Their cortices were rapidly removed, and frozen immediately in liquid nitrogen, and stored at –80 °C.

### 2.1. Western blot analysis

Each sample was homogenized in RIPA buffer (10 μL/mg) containing a 10 μL/mL protease inhibitor cocktail (Halt Protease Inhibitor Single-Use Cocktail, Thermo Scientific) on ice. Lysates were centrifuged at 15,000 × *g* for 15 min at 4 °C and supernatants were removed; protein concentrations were then determined by using a BCA protein assay kit (Novagen, BCA Protein Assay Kit, Merck Millipore, Darmstadt, Germany). Supernatants were then boiled in the sample buffer for 5 min. Then, 20 μg (for Bcl-2) and 60 μg (for cyt-c) protein samples were loaded onto 15% sodium dodecyl sulfate–polyacrylamide gels, separated electrophoretically, and transferred to PVDF membranes (Millipore Corporation, Billerica, MA, USA). The membranes were incubated in blocking buffer (5% milk in Tris-buffered saline [TBS] with 0.1% Tween 20) for 1 h at room temperature, followed by a 1.5 h incubation for Bcl-2 and beta actin, and by an overnight incubation for cyt-c at 4 °C with primary antibodies (Cell Signaling Technology, Bcl-2, #2870, 1:3000; cyt-c #4280, 1:1000; beta actin, Thermo Scientific PA1-46296, 1:2000) Beta actin was used as the loading control. After washing 3 times in TBS containing 0.1% Tween-20, the appropriate secondary antibody (HRP- conjugated IgG) was applied at a 1:5000 dilution for 1.5 h (Cell Signaling Technology, #7074). The membranes were then washed 3 times in TBS. Blots were developed by using Luminata Crescendo Western HRP Substrate (Millipore) and were exposed on the X-ray films. Signals were quantified by gel densitometry scanning in Image J software (Wayne Rasband, National Institutes of Health, Bethesda, MD, USA). Each experiment was repeated 3 times to determine the average value. 

### 2.2. Immunohistochemistry for caspase-3 

Cortex tissues from each group were fixed in 10% neutral formalin for 72 h and processed for paraffin embedding. For immunohistochemical demonstration, sections of 4–5 μm thickness was mounted on polylysine-covered microscope slides. The slides were incubated in a microwave oven in 0.01 M/L citrate buffer (pH: 6.0, Cat: AP-9003-500, Lot: 9003LT13610, LabVision, Fremont, CA, USA). Endogenous peroxidase activity was blocked in 3% hydrogen peroxide (Cat: TA-125-HP, Lot: HP23491, Thermo, Fremont, CA, USA). Epitopes were stabilized by application of serum blocking solution (Ultra V Block Cat: TA-125-HL, Lot: RHL-120705, Thermo); slides were then incubated with anticaspase-3 primary antibody (RB-1197-P, Lot: 11010N, Thermo) for 45 min at room temperature. The biotinylated secondary antibody (Cat: TA-125-HL, Lot: RHL-120705, Thermo) streptavidin–peroxidase (Cat: TA-125-HL, Lot: RHL-120705, Thermo) was applied to the slides. 3-Amino-9-ethylcarbazole (Cat: TA-125-SA, Lot: ASA130503, Thermo) was used as a chromogen. Counterstaining was done using Mayer’s hematoxylin (Cat: TA-125-MH, Lot: AMH70809, LabVision, Fremont, CA, USA). Sections were evaluated in the Leica Q Vin 3 program by obtaining images with a Leica DM 4000 (Wetzlar, Germany) computer-supported imaging system.

The distribution and intensity patterns of caspase-3 immunohistochemical staining were evaluated using H-score. Serial sections were examined and immunolabeling patterns were compared. The labeling intensities were graded semiquantitatively and the H-score was calculated by using the following equation: H-SCORE = ∑Pi (i + 1), where i = intensity of labeling with a value of 1, 2, or 3 (weak, moderate, or strong, respectively), and Pi is the percentage of labeled cells for each intensity, varying from 0% to 100% [30].

### 2.3. Statistical analysis

Data are represented as mean ± S.E.M. Statistical analysis was performed with the one-way analysis of variance (ANOVA) with Fisher’s LSD post hoc test. A difference with P < 0.05 was considered statistically significant.

## 3. Results 

### 3.1. Western blotting 

**Figure 1:**
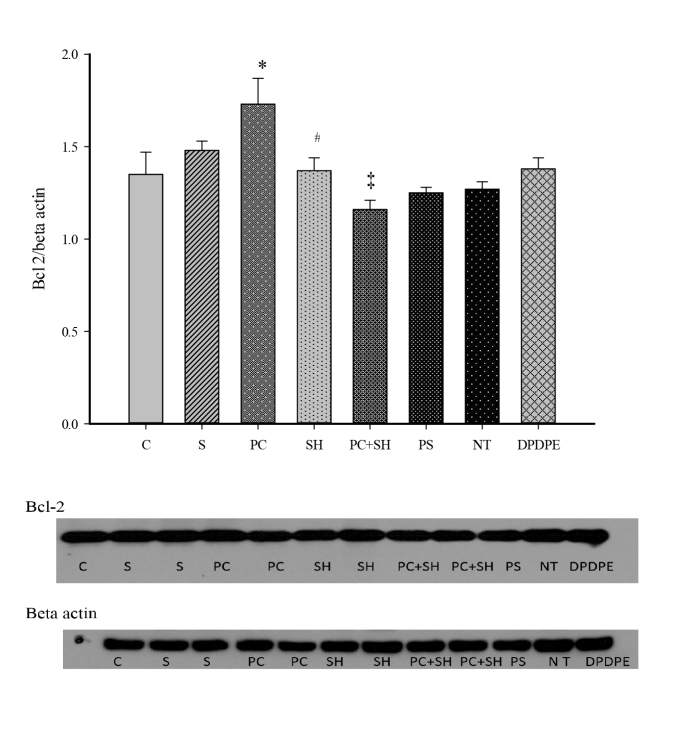
Bcl-2 (26 kD) expressions (n = 6). 20 μg protein was loaded in each well. Beta actin (46 kD) was used as loading control. * C vs PC: P < 0.05, Bcl-2 levels were increased in PC group. ‡ PC vs PC + SH, P < 0.001; Bcl-2 levels were decreased in the PC + SH group. # PC vs SH, P < 0.05; Bcl-2 levels were decreased in the severe hypoxia group.

**Figure 2:**
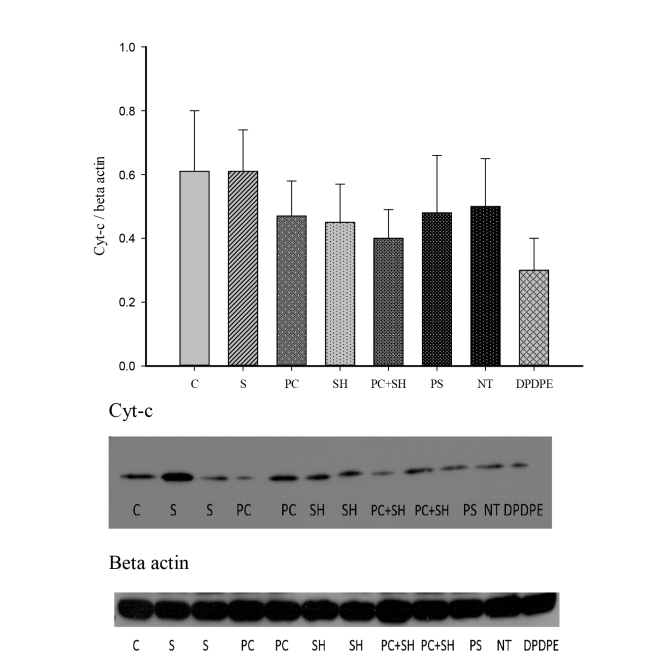
Demonstration of Cyt-c (14 kD) expressions; 60 μg protein was loaded in each well. Beta actin was used as loading control (46 kD). (n numbers for C: 4, S: 6, PC: 5, SH: 6, PC + SH: 6, PS: 3, NT and DPDPE: 4).

The differences in the mean values among groups for the Bcl-2 levels were significant (ANOVA F: 4.810, P < 0.001). Bcl-2 expressions in the hypoxic preconditioning group were significantly higher than in the control (P < 0.05), severe hypoxia (P < 0.05), and PC + SH groups (P < 0.05) (Figure 1). Cyt-c expressions were not significantly different among the groups (ANOVA F: 0.662, P > 0.05) (Figure 2). DPDPE and NT had no significant effect on the Bcl-2 and cyt-c expressions (Figures 1 and 2). 

### 3.2. Immunohistochemistry

**Figure 3:**
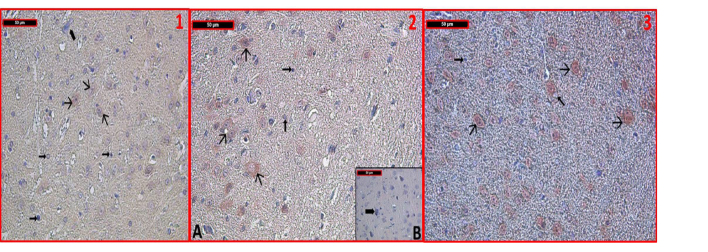
In the control group, neurons are seen in their normal histological structures. While caspase-3 immunoreactivity is significantly weak in some neurons, immunoreactivity in glial cells has not been determined (1). In the sham group, caspase-3 immunoreactivity appears to be weak in neurons (2A). In some areas, neurons with degenerated nucleus were distinguished (2B). When compared to the sham group, caspase-3 immunoreactivity was increased in the hypoxic preconditioning group. Involvement changed from weak to moderate. In this group, positive immunoreactive glial cells were also relatively increased (3). (→): positive caspase-3 immunoreactive cells, (Æ): negative caspase 3 immunoreactive cells (immune peroxidase–hematoxylin ×400; n = 6 for each group).

**Figure 4:**
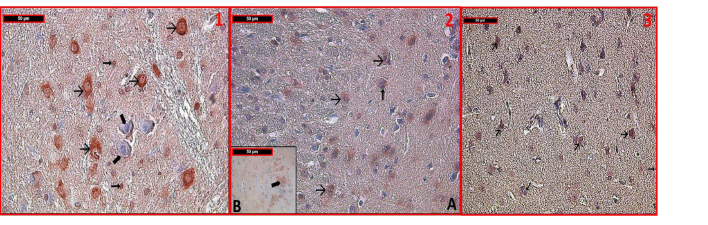
Involvement of caspase-3 immunostaining in the severe hypoxia group was significantly strong in some neurons. In contrast to the other groups, caspase-3 immunoreactivity was also seen in glial cells (1). In the PC + SH group, the involvement of caspase-3 immunoreactivity decreased when compared to the severe hypoxia group. Immunoreactive cells were shown at different cortical regions in 2A and 2B. Involvement of caspase-3 immunostaining of PS group was found to be similar to that of the PC + SH group in both neurons and glial cells (3). (→): positive caspase-3 immunoreactive cells, (Æ): negative caspase 3 immunoreactive cells (immune peroxidase–hematoxylin ×400; n = 6 for each group).

**Figure 5:**
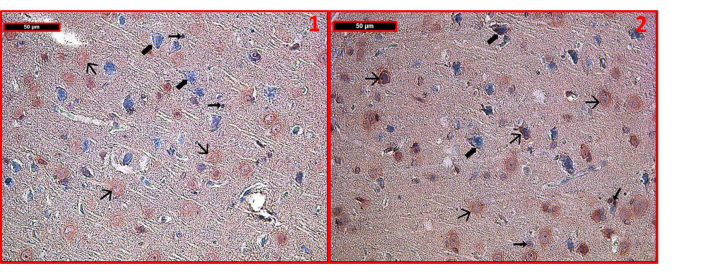
When compared to the severe hypoxia group, caspase-3 involvement was decreased in the DPDPE group. However, in this group, some glia cells showed caspase-3 immunoreactivity as in the severe hypoxia group (1). Caspase-3 involvement in the NT group was increased compared to that of the DPDPE group, and caspase-3 immunoreactivity was found to be similar to that of the severe hypoxia group. Unlike the severe hypoxia group, condensations in the nucleus and cytoplasm were noticed in some neurons (2). (→): positive caspase-3 immunoreactive cells, (Æ): negative caspase 3 immunoreactive cells (immune peroxidase–hematoxylin ×400; n = 6 for each group).

The differences in the mean values of H-scores amongst the groups are significant (ANOVA F: 673.035, P < 0.001). Cortical neurons and glial cells had normal histological structures in the control group (Figure 3). Caspase-3 immunoreactivity of these cells was also quite low. In the sham group (Figure 3), there were some neurons distinguished with degenerated nuclei; these neurons showed higher caspase-3 immunoreactivity than the control group. However, when all of the tissue samples were analyzed, there were no significant differences between the control and sham groups in terms of caspase-3 immunoreactivity. Caspase-3 immunoreactivity of cortical neurons and glial cells in the severe hypoxia (Figure 4) and NT (Figure 5) groups was higher than in the control and the hypoxic preconditioning groups (Figure 3) (P < 0.001). As in the PC + SH group (Figure 4), in the PS group, caspase 3 immunoreactivity of cortical neurons and glial cells was lower than in the NT group (P < 0.001) (Figure 4).

**Figure 6:**
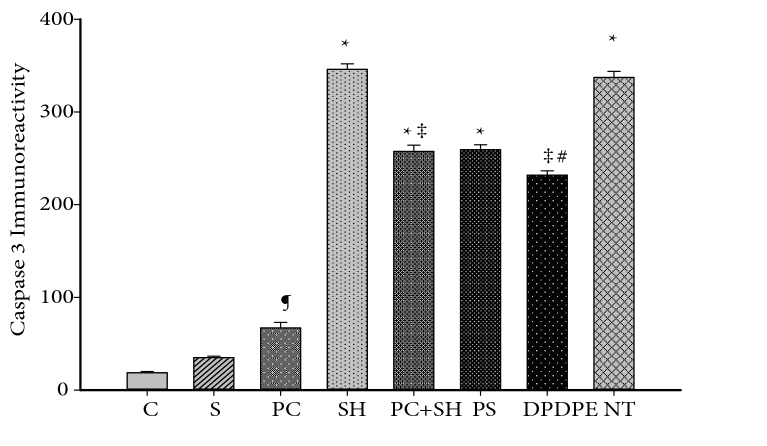
H scores of caspase-3 immunoreactivity. ¶ Immunoreactivity of PC group was higher than in the C and S groups. *Compared with the C, S, and PC groups, immunoreactivity was higher in the SH, PC + SH, PS, NT groups (P < 0.001). ‡ The PC + SH and DPDPE groups have lower caspase-3 immunoreactivity than SH and NT groups (P < 0.001). #Levels of caspase-3 immunoreactivity were lower in the DPDPE group than in the PC + SH group (P < 0.001).

There was no significant difference between the severe hypoxia and NT groups. Strong caspase-3 immunoreactivity was seen in both groups. The caspase-3 immunoreactivity of neurons and glial cells in the PC + SH and DPDPE groups (Figure 5) was lower than in the severe hypoxia and NT groups (P < 0.001), but not lower than in the control (Figure 3), hypoxic preconditioning, and sham groups. Caspase-3 immunoreactivity in the DPDPE group was lower than in the PC + SH group (P < 0.001) (Figure 6).

## 4. Discussion

Our results in this study showed that in the presence of hypoxic preconditioning, DOR agonist DPDPE may protect cortical neurons by reducing caspase-3 immunoreactivity triggered by severe hypoxia. Since the frontal cortex is one of the most vulnerable areas in the brain [31,32], we investigated the effect of delta opioid receptors on hypoxic preconditioning-mediated neuroprotection in the frontal cortex. 

Hypoxic preconditioning is known to have a protective effect on vital organs such as the heart and brain. Short-term protective effects of preconditioning (minutes to hours) include activation and posttranslational modification of existing proteins. However, long-term effects (hours to days) of it are related to the synthesis of new proteins. In several hypoxic preconditioning models, DOR receptors have been shown to have protective effects on heart and brain tissues in particular. These protective effects of DORs are related with ionic homeostasis, neuronal conductance, neurotransmitter release, and regulation of apoptotic and proapoptotic signal pathways. The DORs-G protein-PKC-pERK-Bcl-2 pathway is the most important pathway in opiodergic neuroprotection; in several studies, hypoxic preconditioning has been shown to activate this pathway [9,28,33,34]. In our study, the antiapoptotic protein Bcl-2 levels increased with hypoxic preconditioning; this finding is consistent with studies showing increased Bcl-2 levels through hypoxic preconditioning [35–39]. 

However, the increase in Bcl-2 levels was not observed in the groups that were treated with both preconditioning and severe hypoxia together. Treatments of DOR agonist or antagonist also did not show statistically significant effects of Bcl-2 and cyt-c levels in these groups. In severe hypoxia and anoxia conditions, cells activate a series of pathways leading to apoptosis. The initiation of apoptosis depends on the ratio between activator and inhibitor molecules. Therefore, the Bax/Bcl-2 ratio is an important determinant for initiating apoptosis [40]. Rybnikova et al. [41] showed that severe hypoxia did not affect the Bcl-2 levels in the hippocampus and neocortex, while proapoptotic protein Bax levels were increased by severe hypoxia. Therefore, they suggested that increased Bax/Bcl-2–Bcl-xL ratio triggered neuronal apoptosis. In the present study, we did not measure Bax levels or the Bax/Bcl-2 ratio; this is one of the limitations of our study. The first 2–4 hours after hypoxic/ischemic injury are important for cyt-c release from mitochondria and ensuing apoptotic cascades [41,42]. In our study, tissue samples were collected immediately after severe hypoxia administration; this could be the reason for no significant changes in cyt-c levels. Hypoxic/ischemic preconditioning increases the synthesis of antiapoptotic proteins like Bcl-2 and Bcl-xL, while it decreases synthesis of caspase-3, and causes a decrease in p53 activation and cyt-c release from mitochondria [43]. Ma et al. [9] have suggested that neuroprotective effects of DORs in hypoxic preconditioning involve the DOR-G protein-PKC-pERK-Bcl-2 signaling pathway; stimulation of this pathway by preconditioning increases levels of protective proteins such as Bcl-2 and inhibits death signals like p38 MAPK-mediated cyt-c release from mitochondria. 

Peng et al. [28] showed that hypoxic preconditioning is neuroprotective against intraocular pressure-induced stress in rat retinas. The protective mechanism here is related to the restoration of redox imbalance and the inhibition of proapoptotic caspase-3 via stimulation of the DOR-ERK pathway. In the present study, caspase-3 immunoreactivity increased significantly in response to severe hypoxia, and this increase was extenuated by hypoxic preconditioning treatment. Moreover, DPDPE treatment after hypoxic preconditioning and before severe hypoxia reduced this increase a bit more. DOR antagonist Naltrindole diminished the effectiveness of hypoxic preconditioning in extenuating the increase of caspase-3 immunoreactivity. This finding is compatible with those of the studies which show that DORs are protective against hypoxia/ischemia-induced apoptotic processes. There have also been some studies which underline the important role of DORs in ionic homeostasis during hypoxic/ischemic conditions [44]. Cellular ionic homeostasis has a well-known substantial effect on the activation of the mitochondria-related intrinsic pathway of apoptosis. In the present study, we did not investigate this pathway. 

In conclusion, our results suggest that cortical cells are resistant to apoptosis via increased expression of Bcl-2 and lowered immunoreactivity of caspase-3 in the cortex, and that DOR is involved in neuroprotection induced by hypoxic preconditioning via caspase-3 pathway in cortical neurons. However, our findings are not sufficient to explain the mechanism of DOR-mediated protection against hypoxic/ischemic insult.

## Acknowledgments

The authors thank Asst. Prof. Dr. Gamze Turna for her technical assistance. This study was supported by Gazi University Committee of Scientific Research (Project No: 01/2010-32).
